# Human Mitragynine and 7-Hydroxymitragynine Pharmacokinetics after Single and Multiple Daily Doses of Oral Encapsulated Dried Kratom Leaf Powder

**DOI:** 10.3390/molecules29050984

**Published:** 2024-02-23

**Authors:** Marilyn A. Huestis, Martin A. Brett, John Bothmer, Ramsey Atallah

**Affiliations:** 1 Institute of Emerging Health Professions, Thomas Jefferson University, Philadelphia, PA 19107, USA; 2 PK Consultant, 50259 Pulheim, Germany; martin.brett@gmx.net; 3 JB Pharma Consulting, 6418PR Heerlen, The Netherlands; john.bothmer@jbpharmaconsult.nl; 4 Della Terra Pharmaceuticals, Atlanta, GA 30309, USA; ramseya@dellaterrapharma.com

**Keywords:** mass spectrometry, metabolism, analytical toxicology, mitragynine, kratom, 7-hydroxymitragynine

## Abstract

Kratom leaves, consumed by millions worldwide as tea or ground leaf powder, contain multiple alkaloids, with mitragynine being the most abundant and responsible for most effects. Mitragynine is a partial µ-opioid receptor agonist and competitive antagonist at κ- and δ-opioid receptors; however, unlike morphine, it does not activate the β-arrestin-2 respiratory depression pathway. Due to few human mitragynine data, the largest randomized, between-subject, double-blind, placebo-controlled, dose-escalation study of 500–4000 mg dried kratom leaf powder (6.65–53.2 mg mitragynine) was conducted. LC-MS/MS mitragynine and 7-hydroxymitragynine plasma concentrations were obtained after single and 15 daily doses. Mitragynine and 7-hydroxymitragynine C_max_ increased dose proportionally, and AUC was slightly more than dose proportional. The median mitragynine T_max_ was 1.0–1.3 h after single and 1.0–1.7 h after multiple doses; for 7-hydroxymitragynine T_max_, it was 1.2–1.8 h and 1.3–2.0 h. Steady-state mitragynine concentrations were reached in 8–9 days and 7-hydroxymitragynine within 7 days. The highest mean mitragynine T_1/2_ was 43.4 h after one and 67.9 h after multiple doses, and, for 7-hydroxymitragynine, it was 4.7 and 24.7 h. The mean 7-hydroxy-mitragynine/mitragynine concentration ratios were 0.20–0.31 after a single dose and decreased (0.15–0.21) after multiple doses. These mitragynine and 7-hydroxymitragynine data provide guidance for future clinical kratom dosing studies and an interpretation of clinical and forensic mitragynine and 7-hydroxymitragynine concentrations.

## 1. Introduction

The kratom (*Mitragyna speciosa* Korth.) tree is a Southeast Asian tropical evergreen in the Rubiacaeae family related to the coffee tree [1]. The oral consumption of kratom leaves dates back hundreds of years in Southeast Asia, where the leaves are typically chewed fresh, brewed into a tea, or processed into tar-like extracts [2]. Millions consume kratom worldwide as food, dried leaf powders in capsules, concocted into beverages or teas, or as processed extracts [3,4,5].

**Figure 1:**
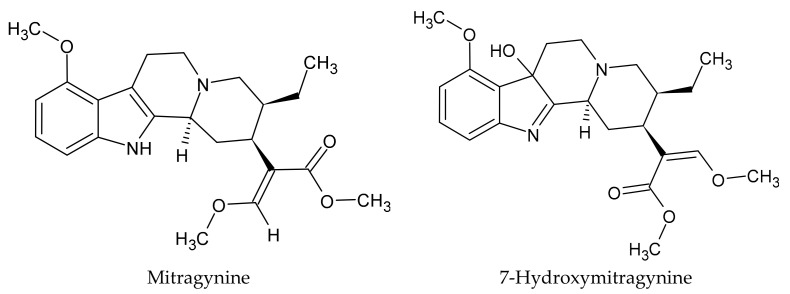
Chemical structures of mitragynine and 7-hydroxy-mitragynine.

Multiple alkaloids are present in kratom including mitragynine, speciogynine, speciociliatine, paynantheine, and other indoles and oxindoles [6,7,8], with their content varying based on geographic location, seasonal variation, and plant part used [9,10,11]. Mitragynine (IUPAC methyl (16E)-9,17-dimethoxy-16,17-didehydro-20β-corynan-16-carboxylate) is the most abundant alkaloid present in kratom and is responsible for many of its effects, although research into the contributions of kratom’s other alkaloids is increasing [12,13,14,15]. Mitragynine is metabolized to 7-hydroxymitragynine (IUPAC methyl (2E)-2-[(2S,3S,7aS,12bS)-3-ethyl-7a-hydroxy-8-methoxy-1,2,3,4,6,7,7a,12b-octahydroindolo[2,3-a]quinolizin-2-yl]-3-methoxyprop-2-enoate), which is not found in measurable amounts in fresh kratom leaves [10,16]. Chemical structures for both mitragynine and its metabolite 7-hydroxymitragynine are shown in Figure 1. Mitragynine and 7-hydroxymitragynine are partial agonists of the human µ-opioid receptor and competitive antagonists at κ- and δ-opioid receptors [17]. Mitragynine has a lower affinity and potency than morphine at the µ-opioid receptor and is unable to induce comparable phosphorylation and GTPγS stimulation [18]. Unlike full µ-opioid agonists, such as morphine and fentanyl, mitragynine does not activate the β-arrestin-2 pathway implicated in the adverse effects of µ-opioid agonists including respiratory depression and constipation [19,20]. Reduced respiratory depression of mitragynine compared to codeine was reported more than 50 years ago [14].

Furthermore, mitragynine and 7-hydroxymitragynine are also bound to adrenergic (α_1_ and α_2_) and serotonergic (5-HT_1A_ and 5-HT_2B_) receptors, producing a different profile of effects than prototypical opioids [16,21,22,23]. Currently, based on this unique pharmacology, there is substantial interest in the development of synthetic medicinal compounds built on the chemical scaffolding of kratom alkaloids for analgesia, namely, for the treatment of opioid withdrawal and opioid use disorder [17,19,24].

Despite the widespread availability and use of kratom, there are few controlled administration studies of mitragynine and 7-hydroxymitragynine exposure from well-characterized kratom products. In the only controlled kratom administration study prior to 2022, plasma mitragynine concentrations were determined in 10 chronic male kratom users after drinking different low kratom tea doses for 7 days [25]. More recently, mitragynine and 7-hydroxymitragynine pharmacokinetics were reported in five participants over 120 h after a single 39 mg mitragynine dose in 2 g kratom tea [26]. These authors also investigated the drug interactions of the same single kratom dose to 12 subjects [27].

Due to the dearth of controlled mitragynine and 7-hydroxymitragynine concentration data and the increasing intake of kratom around the world, pharmacokinetic data are needed for designing future kratom studies and for interpreting mitragynine and 7-hydroxymitragynine concentrations in clinical and forensic investigations. The primary objective of this study was to determine the pharmacokinetics of single doses (SDs), and, for the first time, 15 consecutive daily oral ascending doses (MDs) of dried kratom leaf powder in healthy adults. Pharmacokinetic data obtained after single and multiple dosing included the following: the time course of mitragynine and 7-hydroxymitragynine concentrations over 10 days after a single dose and 23 days after multiple dosing, dose-proportionality, times to reach steady state, half-lives and 7-hydroxy-mitragynine/mitragynine ratios.

## 2. Results

### 2.1. Participants

Twelve participants received active leaf powder (except 13 for the highest dose, due to one participant being replaced). A minimum of one SD or one MD pharmacokinetic parameter, in most cases, the C_max_, was required for inclusion in the SD or MD pharmacokinetic dataset. If participants voluntarily withdrew or were withdrawn from the study due to adverse events, or failed to follow study protocols and were withdrawn during the SD or MD phases of the study, they did not have MD pharmacokinetic data. The pharmacokinetic study for MD occurred after the last MD, resulting in a smaller number of subjects with MD pharmacokinetic data. Table 1 includes participants‘ demographic data.

### 2.2. Bioanalysis

**Figure 2:**
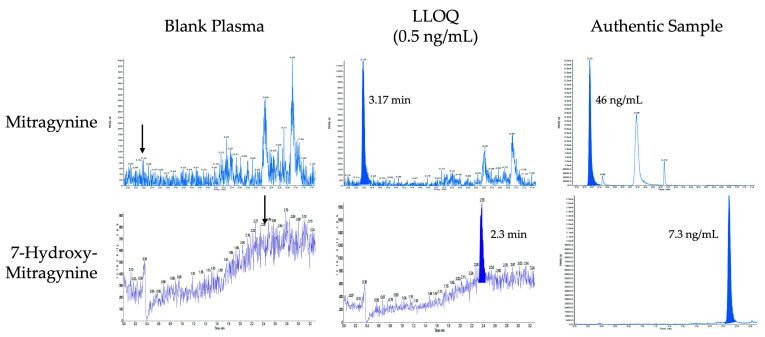
LC-MS/MS chromatograms of blank plasma, plasma fortified at the lower limit of quantification (0.5 ng/mL) for mitragynine and 7-hydroxymitragynine and an authentic plasma sample collected at 1.33 h after the participant received 13.3 mg of mitragynine in 1000 mg of dried kratom leaf, achieving plasma concentrations of 46 ng/mL of mitragynine and 7.3 ng/mL of 7-hydroxymitragynine. Arrows in the blank chromatogram indicate retention time of mitragynine and 7-hydroxymitragynine.

The analytical method for mitragynine and 7-hydroxymitragynine was fully validated according to the FDA Guidance for Industry (May 2018) and EMA Guideline on Bioanalytical Method Validation EMEA/CHMP/EWP/192217/2009 Rev.1 Corr. 2, effective February 2012. Blood was collected with K_2_-EDTA anticoagulant and drawn at specified time points before centrifuging and removing plasma within 2 h. The sample pretreatment consisted of protein precipitation of 100 µL of plasma, and the internal standards were mitragynine-D3 and 7-hydroxymitragynine-D3. Mitragynine and 7-hydroxymitragynine were identified and quantified using reversed-phase ultra-high-performance liquid chromatography/tandem mass spectrometry with MS/MS detection. Carryover was not observed at the highest calibrator concentration. Table 2 includes the most important validation data for mitragynine and 7-hydroxymitragynine. Figure 2 includes LC-MS/MS chromatograms of a blank, the lower limit of quantification, and an authentic plasma sample collected at 1.33 h after one participant received 13.3 mg mitragynine in 1000 mg of dried kratom leaf, achieving 46 ng/mL mitragynine and 7.3 ng/mL 7-hydroxymitragynine plasma concentrations.

### 2.3. Pharmacokinetics

**Figure 3:**
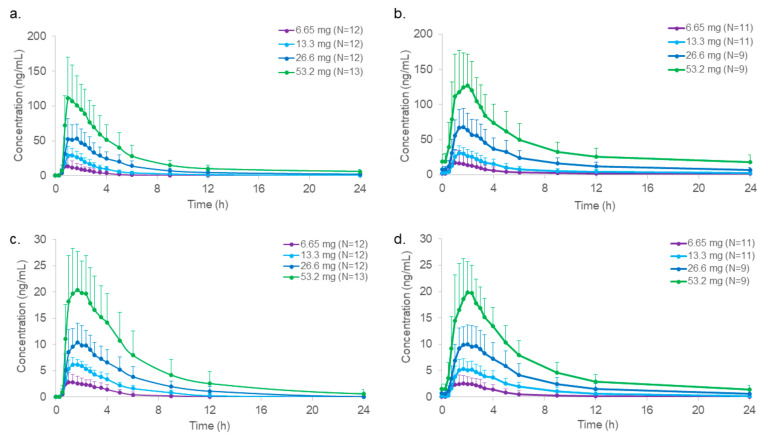
Mean plasma concentration profiles of (**a**) mitragynine (including standard deviation bars) after a single dose (SD); (**b**) mitragynine after 15 multiple doses (MD); (**c**) 7-hydroxymitragynine after an SD; (**d**) 7-hydroxymitragynine after 15 MD mitragynine for all four dried kratom leaf powder doses. Each dose was administered to a different cohort of healthy male and female subjects.

Figure 3 illustrates the mean ± standard deviation plasma concentration profiles of mitragynine and 7-hydroxymitragynine after SD and MD oral 500, 1000, 2000, and 4000 mg encapsulated dried kratom leaf powder doses containing 6.65, 13.3, 26.6, and 53.2 mg of mitragynine, respectively.

Table 3 contains mean (standard deviation) and median (range) plasma mitragynine pharmacokinetic parameters after SD and MD kratom leaf powder. C_max_ and AUC increased with each SD and MD escalation. The SD median T_max_ was fairly consistent from 1.0 to 1.3 h over these doses and similar to mitragynine MD 1.0–1.7 h T_max_. The median terminal half-life and time to last quantifiable concentration generally increased with the dose since plasma concentrations were detectable over a longer period. The highest mitragynine median T_1/2_ was 42.9 h after the highest 53.2 mg SD, and 61.2 h after the 26.2 mg MD. Fluctuation of the mitragynine concentrations across the dosing interval after MD at steady state ranged from 3.3 to 5.6, with lower values observed at higher doses. Dose proportionality of mitragynine was demonstrated based on C_max_ and C_max_,_ss_ during SD and MD. However, AUC_0-Tlast_ and AUC_0-tau,ss_ did not fulfill the proportionality criterion, with increases slightly higher (1.42 SD and 1.33 MD) than dose proportionality predicted.

The mean (standard deviation) and median (range) plasma pharmacokinetic parameters for 7-hydroxymitragynine after SD and MD oral mitragynine in kratom leaf powder are shown in Table 4. 7-hydroxymitragynine C_max_ and AUC were lower than those of mitragynine but also increased in a dose-appropriate manner after SD and MD. The median 7-hydroxymitragynine T_max_ was similar to mitragynine at 1.2–1.8 h and 1.3–2.0 h after SD and MD, respectively. Apparent increases in median T_1/2_ were seen with increasing doses as for mitragynine, with a median (range) T_1/2_ of 4.0 h (1.7–11.4) and 9.1 h (2.2–71.6) after the highest SD and MD, respectively. 7-hydroxymitragynine MD fluctuation was 3.4–9.8, with lower values at higher doses. 7-hydroxymitragynine dose proportionality was confirmed based on C_max_ and C_max,ss_ after SD and MD but was slightly greater than dose-proportional (1.18-fold) based on AUC_0-Tlast_ after SD and 1.32-fold after MD based on AUC_0-tau,ss_. Multiple concentration peaks were observed in some participants more than 4 h after SD and MD, suggesting that food consumption 4 h after kratom leaf powder dosing might have contributed to these later concentration peaks.

### 2.4. Ratios of 7-Hydroxymitragynine/Mitragynine Plasma Concentrations

**Figure 4:**
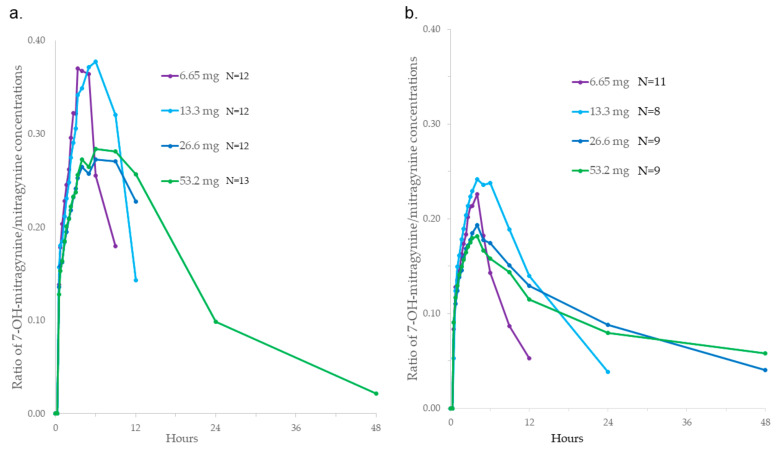
Mean ratios of 7-hydroxymitragynine/mitragynine plasma concentrations after (**a**) single (SD) and (**b**) multiple (MD) oral 6.65–53.2 mg mitragynine doses in dried kratom leaf powder.

7-hydroxymitragynine/mitragynine concentration ratios are shown in Figure 4. Mean ratios were 0.20–0.29 based on C_max_ and 0.21–0.31 based on AUC_0-24_ after a SD, with the highest ratios observed after the lowest 6.65 mg mitragynine dose. A similar pattern was seen with MD, with mean ratios of 0.16–0.21 based on C_max,ss_ and 0.15–0.18 based on AUC_0-tau,ss_. The ratio was generally higher after SD compared to MD and higher at the lower doses.

### 2.5. Accumulation of Mitragynine and 7-Hydroxymitragynine during MD

Accumulation of mitragynine and 7-hydroxymitragynine was assessed by comparing C_max,ss_ to C_max_ and AUC_0-tau,ss_ to AUC_0-24_ for the MD and SD, respectively. For mitragynine, the accumulation was low to moderate across doses with C_max_ ratios of 1.1–1.3 and AUC ratios of 1.6–1.9. Corresponding ranges for 7-hydroxymitragynine were lower: 0.9–1.0 and 1.0–1.3, indicating no or low 7-hydroxymitragynine accumulation after MD.

### 2.6. Time to Reach Steady State for Mitragynine and 7-Hydroxymitragynine during Multiple Dosing

**Figure 5:**
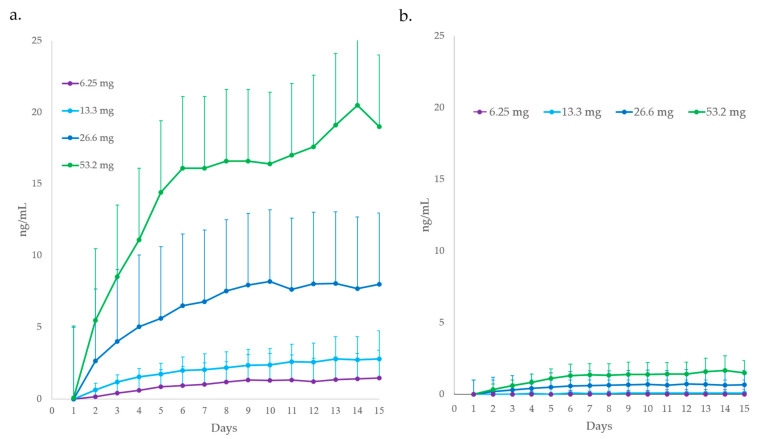
Trough plasma (**a**) mitragynine and (**b**) 7-hydroxy-mitragynine concentrations (including standard deviation bars) on days 1–15 of consecutive 6.65–53.2 mg mitragynine doses in dried kratom leaf powder during the attainment of steady-state concentrations.

Figure 5 illustrates the accumulation in mitragynine and 7-hydroxymitragyine after multiple dosing over four increasing kratom leaf powder doses. Based on the trough concentrations determined each MD day, the time to reach steady state for mitragynine was 8–9 days. For 7-hydroxymitragynine, the time to reach steady state was 7 days based on the two highest doses, as the number of trough samples above the assay quantification limit was insufficient for the two lowest doses.

## 3. Discussion

This study was the first to provide extensive mitragynine and 7-hydroxymitragynine pharmacokinetic data in a controlled setting after increasing kratom leaf powder SD and MD. Intensive blood sampling up to 10 days after SD and 23 days after MD ensured well-characterized pharmacokinetic exposure parameters and terminal half-lives. Concentrations of 7-hydroxy-mitragynine originated primarily from mitragynine metabolism because its content in the kratom leaf powder was <0.01%.

In the current study, median mitragynine T_max_ after SD and MD ranged from 1.0 to 1.7 h, with only slightly longer T_max_ for 7-hydroxymitragynine (1.2–2.0 h). In two recent studies with kratom tea, the median T_max_ for both compounds was 1.0 h [26,27]. The slightly longer T_max_ in the present study may be due to the encapsulated kratom leaf powder formulation. Concentration–time curves for both analytes showed an initial rapid descent after C_max_ for about 6 h and then a much slower terminal phase.

The individual mitragynine SD C_max_ of 16.0–90.0 ng/mL (24 subjects) following 13.3–26.6 mg mitragynine was similar to the range reported by Trakulsrichai [25] of 18.5–105 ng/mL (9 subjects) following 10–23.6 mg mitragynine in kratom tea. However, subjects in the latter study received different low doses (6.3–11.5 mg) of kratom tea for 7 days prior to the pharmacokinetics profile day and were not asked to cease other kratom use. The contribution of this additional kratom consumption to the reported mitragynine concentrations is unclear.

Compared to the recent SD kratom tea investigations [26,27], the current study documented higher mitragynine exposure. The geometric mean C_max_ and AUC_0-24_ were 119 nM (approximately 47.5 ng/mL) and 388 h*nM (approximately 155 h*ng/mL) in six male and six female subjects following 39 mg mitragynine [27]. Our geometric mean results in 12 subjects were 51.0–113 ng/mL for C_max_ and 169–493 h*ng/mL for AUC_0-24_ across 26.6–53.2 mg mitragynine. 7-hydroxymitragynine exposure was similar between the two studies: C_max_ and AUC_0-24_, in the kratom tea study, were 31 nM (approximately 12.9 ng/mL) and 151 h*nM (or approximately 62.7 h*ng/mL) compared to the current study, with 10.2–21.6 ng/mL for C_max_ and 34.2–110 h*ng/mL for AUC_0-24_ across the dose range of 26.6–53.2 mg mitragynine. Differences in the liquid kratom tea and solid encapsulated leaf powder formulations likely impacted absorption.

Between-subject variability for C_max_ and AUC_0-24_ was quite high after SD and MD. For mitragynine, CV% was generally 30–60%, increasing up to 99% at the lowest 6.65 mg dose. For 7-hydroxymitragynine, between-subject variability was slightly lower at 20–55%, with the highest variability of 67%, again, at the lowest dose.

Mitragynine and 7-hydroxymitragynine terminal half-lives generally increased with increasing doses, as concentrations were measurable for longer at higher doses compared to lower doses. However, increases in terminal half-life were also observed between single and multiple dosing, which will be explored in future investigations.

The same issue of low concentrations after lower doses also affected AUC_0-inf_, in some cases underestimating AUC_0-inf_ and overestimating CL/F. For example, the mean CL/F of mitragynine for the lowest single 6.65 mg dose was much higher (278 L/h) than for the higher doses (94–123 L/h).

Accumulation factors for mitragynine comparing C_max,ss_ and AUC_0-tau,ss_ during MD at steady state with C_max_ and AUC_0-24_ after a SD were low to moderate, ranging from 1.1 to 1.3 and 1.6–1.9, respectively. Corresponding ranges of 0.9–1.0 and 1.0–1.3 were observed for 7-hydroxymitragine, indicating no or only low accumulation after MD. These results contradict the observed long terminal T_1/2_, suggesting that higher accumulation ratios should have been found. Sahin and Benet [28] address the issue of the overprediction of accumulation from extended terminal half-lives and define the operational multiple dosing half-life (t_1/2,op_) as being equal to the dosing interval at a steady state, where the maximum concentration at the steady state is twice the maximum concentration found for the first dose. It would be useful to define an operational or MD T_1/2_ that is more relevant than the terminal T_1/2_ for predicting the accumulation of mitragynine and 7-hydroxymitragynine after different dosing regimens.

7-hydroxymitragynine to mitragynine ratios were always higher after SD than MD, and the highest ratios were consistently after lower doses. Across the concentration–time profile, ratios generally increased to a maximum and then decreased over time. The decrease reflects the shorter half-life of the metabolite compared to the parent compound. The range of the median C_max_ ratios observed at the top two doses was 0.18–0.21, which was similar to the median of 0.27 previously reported in a small number of subjects [27].

The CYP3A4 enzyme plays a major role in the metabolism of mitragynine to 7-hydroxymitragynine based on in vitro studies using human liver microsomes and S9 fractions [29]. Since CYP3A enzymes are the predominant phase 1 metabolism enzymes in the liver and intestine of dogs and men [30], it is of interest to compare the metabolic ratios of 7-hydroxymitragynine to mitragynine between dogs and humans. In one nonclinical study [31], 5 mg/kg mitragynine was administered orally to five beagle dogs, from which plasma concentration profiles were obtained for mitragynine and 7-hydroxymitragynine up to 24 h post-dose. The ratios of metabolite to parent compound were 11.3% and 12.6% for C_max_ and AUC, respectively, about one-half of the ratios observed in the current human study.

The limitations of this study include the limited range of doses that were prescribed by the reviewing ethics committee and Health Canada. Future clinical studies will expand the administered dose range. The long terminal mitragynine T_1/2_ requires an adequate washout in pharmacokinetic studies between treatments. After a SD, mitragynine concentrations were measurable in some subjects 10 days after dosing; however, in all cases, concentrations were <5% of C_max_. The FDA guideline [32] recommends a washout of at least five half-lives or until concentrations in each subject are ≤5% of C_max_. Here, a 10-day washout was adequate for a single kratom leaf powder dose containing up to 53.2 mg mitragynine; however, for the MD regimens, at least a 14-day washout period is recommended. After 15 MD, a steady state was reached in 7–9 days for both analytes. Visual inspection of the trough concentrations (Figure 5) suggested that, after day 10, a further slight increase in trough concentrations appears to occur at the highest 53.2 mg dose. It would be prudent to monitor trough concentrations over a longer period for higher doses to confirm steady state attainment.

In conclusion, a wealth of pharmacokinetic data were obtained from this largest kratom leaf powder administration study, providing much-needed new insights into the pharmacokinetic characteristics of mitragynine and 7-hydroxymitragynine over a range of increasing SD and MD. These data are of considerable help for the planning and design of future clinical kratom studies and, importantly, in interpreting clinical mitragynine and 7-hydroxymitragynine concentrations.

## 4. Materials and Methods

### 4.1. Study Design

**Figure 6:**
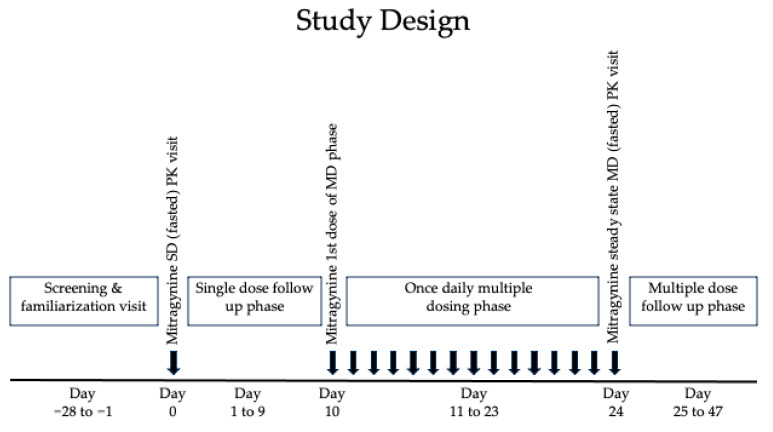
Study design showing dosing and in-person clinical visits (single dose (SD) follow-up phase days 1, 2, 3, 5, 7, and pre-dose day 10; 15 multiple doses (MDs), follow-up phase days 25, 26, 27, 29, 31, 34, 40, and 47), including two intensive pharmacokinetic (PK) days after a single oral mitragynine dose and on the last of 15 consecutive 6.65–53.2 mg daily mitragynine doses of dried kratom leaf powder.

A randomized, between-subject, double-blind, placebo-controlled, dose-escalation, single-site pharmacokinetic study of encapsulated dried kratom leaf powder was carried out for 10 days after SD, 15 days MD, and 23 days follow-up in healthy adults, with 12 participants receiving active kratom leaf powder per dose. There were 31 clinic visits over 47 days; participants fasted (≥10 h) before SD and during the last MD pharmacokinetic visits (Figure 6). Standardized meals were provided 4 and 10 h after the morning dose.

The study was conducted in accordance with the Declaration of Helsinki and approved by the Advarra Institutional Review Board (protocol code 00048457 on 20 June 2022). The Advarra Institutional Review Board is organized and operates in compliance with the US and Canadian regulations and policies governing research with human subjects. Advarra’s IRB is registered with the FDA and OHRP; IRB Organization (IORG) Number: 0000635, FWA Number: 00023875, IRB Registration Number: 00000971. Advarra is fully accredited by the Association for the Accreditation of Human Research Protection Programs (AAHRPP). The Advarra Institutional Review Board and Health Canada approved the study. Informed consent was obtained from all subjects involved in the study.

### 4.2. Study Population

Non-smoking healthy males and females of 18–55 years with BMI ≥ 18.5 and ≤29.9 kg/m^2^ participated. Participants self-reported being either kratom-naïve or had no kratom use for ≥12 months prior to the first study visit. Female participants of childbearing potential had negative pregnancy tests and were not breastfeeding; appropriate birth control was utilized by all participants throughout the study. No medications were permitted within 7 days (14 days for enzyme inducers) or 7 half-lives of day 0, whichever was longer. Randomization was stratified by sex for males/females per dose.

Participants were excluded if there was a known allergy to product ingredients or known genetic polymorphism of CYP4503A4, CYP2D6, or CYP1A2. Fasting was required 10 h prior to the familiarization visit and prior to the pre-dose blood sample on days 0 and 10–24. The presence of gastrointestinal, hepatic, renal, cardiovascular, respiratory, autoimmune, endocrine, neurological, or active psychiatric diseases was exclusionary.

Participants were excluded if there was an acute illness within 2 weeks of the single dose, a positive urine drug test at screening, a history of drug or alcohol abuse in the past 12 months, a Leeds Dependence Questionnaire score ≥ 21, or a consumption of ≥2 alcoholic beverages a day. Positive serology results for HIV, HBsAg, or HCV or a history of cancer were exclusionary. Participants were non-smokers who had not used nicotine patches or gums for >3 months prior to the first dose and had veins suitable for cannulation/repeated venipuncture. Caffeine intake was restricted for 24 h prior to the two pharmacokinetic study days and 12 h prior and 5 h post each visit; alcohol and cannabinoids were restricted for 24 h prior to and 12 h post days 1–25 and day 47. Hepatic or renal dysfunction with ALT or AST ≥ 2X upper limit of normal (ULN) and serum creatinine or BUN ≥ 1.5X ULN were evaluated by the principal investigator. Participation in another research study or blood donation within four (4) weeks prior to single dosing was prohibited.

### 4.3. Dosing

The kratom dried leaf powder (MitraLeaf^TM^) was manufactured by Johnson Foods, LLC., Kratom capsules were labeled according to ICH-GCP and Health Canada guidelines and stored at room temperature (20–25 °C) with relative humidity ≤65%. A large batch of dried kratom leaf was mixed in an industrial powder mixer to homogenize the powder. The powder was weighed and encapsulated, and quality control was performed to ensure adequate weight in the capsules. To determine the concentration of mitragynine in each capsule, Avomeen, the laboratory performing the LC-MS/MS analysis of the kratom powder, emptied 50 capsules of powder, mixed the powder well, and weighed an aliquot of the powder for analysis. The powder was extracted and analyzed using LC-MS/MS for mitragynine and 7-hydroxymitragynine. The Avomeen Certificate of Analysis showed 13.3 mg mitragynine in 1000 mg of the dried kratom leaf powder. Four cohorts of 12 different subjects received one of four increasing doses of 6.65 mg, 13.3 mg, 26.6 mg, and 53.2 mg mitragynine contained in 500, 1000, 2000, or 4000 mg leaf powder in 1, 2, 4, or 8 capsules, respectively, and 6 subjects at each dose received placebo. After 10 days of monitoring after the SD, 15 consecutive ascending doses of 6.65 mg, 13.3 mg, 26.6 mg, and 53.2 mg mitragynine in 500, 1000, 2000, or 4000 mg of leaf powder were administered, respectively, to participants. 7-hydroxymitragynine concentrations were <0.01% kratom leaf powder. Interim safety analyses included laboratory assessments (hemoglobin, liver, and kidney function tests, adverse events (AEs), and an assessment of the Clinical Opiate Withdrawal Scale (COWS) and Subjective Opiate Withdrawal Scale (SOWS) after the completion of each dose cohort prior to the decision by medical staff to proceed to the next higher dose).

### 4.4. Blood Collection Timeline for Single and Multiple Dose Pharmacokinetic Study

Blood collections (total 484 mL) for the mitragynine and 7-hydroxymitragynine concentrations occurred prior to and 0.25, 0.5, 0.75, 1, 1.33, 1.67, 2, 2.33, 2.67, 3, 3.5, 4, 5, 6, 9, and 12 h after the single dose and after the 15th multiple dose. Participants returned to the clinic 24, 48, and 72 h—and 5, 7, and 10 days—after the SD for the daily post-dose blood collection. Participants fasted for ≥10 h prior to the pre-dose blood collection. Standardized meals were provided 4 and 10 h after the morning dose. Follow-up visits for MD occurred 1, 2, 3, 5, 7, 10, 16, and 23 days later, with a single blood collection in the morning. The follow-up phase began 24 h after the last MD, day 25, and ended on day 47 (end of study, EOS).

### 4.5. Pharmacokinetics

Pharmacokinetic parameters utilized standard non-compartmental methods (SAS version 9.4). Peak concentration (C_max_/C_max,ss_), time to reach C_max_ (T_max/_T_max,ss_), time of last measurable concentration (T_last_/T_last,ss_), area-under-the-curve (AUC) from dosing to 24 h (AUC_0-24h_/AUC_0-tau,ss_) and 72 h (AUC_0-72_), last observable concentration (AUC_0-Tlast/_AUC_0-Tlast,ss_), infinity (AUC_inf_), terminal phase half-life (T_1/2_/T_1/2_,_ss_), terminal phase rate constant (K_el_/K_el,ss_), apparent total clearance (CL/F/CL/F_,ss_), and ratio 7-hydroxymitragynine/mitragynine concentrations were determined. During MD, minimum concentration (C_min,ss_), average concentration (C_av,ss_), trough concentration (C_trough_), and fluctuation ((C_max,ss_ − C_min,ss_)/C_av,ss_) during the dosing interval were also quantified. Accumulation of mitragynine and 7-hydroxymitragynine with MD was compared to SD AUC and C_max_ at each dose.

### 4.6. Statistical Analysis

Analysis sets included all participants with sufficient data for the calculation of at least one pharmacokinetic parameter after SD or MD (SAS version 9.4). A power model for C_max_ or AUC_0-T_ after SD and C_max,ss_ or AUC_0-tau_ after MD investigated dose proportionality. The power model was pharmacokinetics parameter = α*dose^β^. A regression analysis was conducted using the log form of this equation: ln (pharmacokinetics parameter) = lnα + β ∗ ln(dose) + *ε*. Estimates for α and the slope β were obtained, with a 90% confidence interval calculated for β. The criterion for dose proportionality was sufficiently met if this 90% confidence interval was entirely contained within the interval (1 + [ln(0.5)/ln(*r*)], 1 + [ln(2)/ln(*r*)]), where r is the ratio of highest dose divided by the lowest dose. The time required for mitragynine and 7-hydroxymitragynine to reach a steady state was estimated using a Helmert coding approach. The model included the day as a fixed effect, the subject as a random effect, and the logarithm of the concentration as a response variable. Pair-wise contrasts were then estimated, enabling an assessment of the time to reach steady state.

## Figures and Tables

**Table 1:** Participants’ demographic data.

Mitragynine	6.65 mg		13.3 mg		26.6 mg		53.2 mg	
Dried Kratom Leaf Powder	500 mg		1000 mg		2000 mg		4000 mg	
	SD	MD	SD	MD	SD	MD	SD	MD
# Subjects	12	11	12	8	12	9	13	9
Gender	6 F 6 M	6 F 5 M	4 F 8 M	3 F 5 M	4 F 8 M	3 F 6 M	7 F 6 M	4 F 5 M
Race								
AI	0	0	0	0	0	0	2	2
Asian	2	2	5	3	1	0	0	0
Black	0	0	0	0	4	2	1	1
Unknown	0	0	0	0	0	0	1	1
White	10	9	7	5	7	7	9	5
Ethnicity	2 H 10 NH	2 H 9 NH	1 H 11 NH	1 H 7 NH	0 H 12 NH	0 H 9 NH	3 H 10 NH	3 H 6 NH
Mean age (STD) years	30.8 (9.3)	31.3 (9.5)	32.9 (9.4)	33.1 (9.8)	35.8 (11.4)	38.4 (12.2)	33.2 (9.6)	32.4 (7.8)
BMI Kg/m^2^	23.1 (2.7)	22.8 (2.6)	25.3 (2.6)	25.3 (2.7)	26.2 (3.4)	25.7 (3.8)	23.4 (2.9)	23.9 (3.1)

SD, single-dose phase; MD, multiple-dose phase; AI, American Indian or Alaska Native; H, Hispanic; NH, not Hispanic; STD, standard deviation; BMI, body mass index.

**Table 2:** Validation data for the LC-MS/MS quantification of mitragynine and 7-hydroxymiragynine in human plasma.

Validation Parameter	Mitragynine	7-Hydroxymitragynine
Linearity	0.5–500 ng/mL	0.5–100 ng/mL
Lower limit of quantification	0.5 ng/mL	0.5 ng/mL
Quality control (QC) concentrations	0.5, 1.5, 250, and 375 ng/mL	0.5, 1.5, 50, and 75 ng/mL
Within-run accuracy (% bias)	−11.7–11.2%	−9.7–13.6%
Within-run precision (%CV)	1.4–10%	1.2–7.4%
Between-run accuracy (% bias)	−0.6–2.2%	2.4–10.4%
Between-run precision (%CV)	3.9–9.3%	4.2–10%
Internal standard normalized matrix factors for low and high QC	1.00–1.02 (CV 0.7–2.2%)	0.995–1.04 (CV 1.0–2.0%)
10-fold dilution integrity %bias (%CV)	−1.7% (3.0%)	−3.0 (2.8%)
Plasma stability for low and high QC		
22 °C for 23 h (% bias)	−0.6–4.4%	4.4–5.8%
−20 °C for 404 days (% bias)	6.7–9.1%	−1.5–−3.1%
−80 °C for 404 days (% bias)	8.4–8.9%	9.2–11.6%
4 freeze-thaw cycles (% bias)	0.4–5.3%	4.6–10.3%
Autosampler stability 4 °C 241 h (% bias)	−2.9–0.1%	−2.9–−3.8%

**Table 3:** Mean (standard deviation) and median (range) plasma pharmacokinetic parameters for mitragynine after administration of increasing single (SD) and 15 multiple doses (MD) of mitragynine in dried kratom leaf powder.

		Cohort 1 6.65 mg	Cohort 2 13.3 mg	Cohort 3 26.6 mg	Cohort 4 53.2 mg
Single dose		*n* = 12	*n* = 12	*n* = 12	*n* = 13
C_max_ (ng/mL)	mean (STD)	17.1 (15.2)	32.1 (12.2)	57.0 (25.1)	125 (51.8)
	median	12.1	29.0	57.4	130
	(range)	(1.8–61.5)	(16.0–58.1)	(20.4–90.0)	(34.2–204)
T_max_ (h)	median	1.0	1.3	1.3	1.3
	(range)	(0.8–2.7)	(0.8–1.7)	(0.8–5.0)	(0.8–2.0)
T_last_ (h)	median	12.0	23.4	71.6	120
	(range)	(4.0–71.0)	(12.0–72.2)	(0.8–120)	(23.7–169)
AUC_0-24_ (h*ng/mL)	mean (STD)	41.4 (32.4)	108 (40.6)	244 (121)	558 (250)
	median	30.6	101	280	648
	(range)	(3.6–111)	(52.8–183)	(3.3–375)	(155–970)
AUC_0-Tlast_ (h*ng/mL)	mean (STD)	45.8 (42.2)	120 (57.7)	305 (164)	837 (466)
	median	30.6	101	357	839
	(range)	(3.6–137)	(52.8–237)	(3.3–480)	(179–1746)
AUC_0-inf_ (h*ng/mL)	mean (STD)	52.8 (51.1)	136 (72.3)	364 ^1^ (154)	908 (520)
	median	34.8	108	417 ^1^	903
	(range)	(4.7–162)	(58.1–288)	(98.4–517)	(191–1951)
AUC_0-Tlast/_	median	0.91	0.92	0.92	0.93
AUC_0-inf_	(range)	(0.74–0.96)	(0.76–0.95)	(0.87–0.95)	(0.88–0.96)
T_1/2 _ (h)	mean (STD)	8.5 (11.8)	15.8 (16.3)	29.3 ^1^ (17.5)	43.4 (23.3)
	median	3.7	10.5	28.2 ^1^	42.9
	(range)	(1.5–35.0)	(2.4–53.9)	(4.4–55.2)	(8.3–84.5)
CL/F (L/h)	mean (STD)	278 (369)	123 (58.1)	102 ^1^ (79.8)	94.0 (84.1)
	median	191	123	63.7 ^1^	58.9
	(range)	(40.9–1424)	(46.2–229)	(51.4–270)	(27.3–278)
Vz/F (L)	mean (STD)	1349 (808)	1892 (1013)	2875 (871)	3788 (1287)
	median	1017	1786	2544	3314
	(range)	(549–2998)	(615–4438)	(1733–4084)	(2104–6173)
Multiple doses		*n* = 11	*n* = 8	*n* = 9	(*n* = 9)
C_max,ss_ (ng/mL)	mean (STD)	21.4 (18.9)	33.5 (7.43)	74.6 (22.8)	143 (50.4)
	median	16.2	35.1	73.0	155
	(range)	(2.8–60.1)	(21.8–43.1)	(42.4–109)	(64.3–215)
T_max,ss_ (h)	median	1.0	1.7	1.7	1.7
	(range)	(0.8–2.3)	(1.0–4.0)	(1.0–3.0)	(1.0–3.0)
T_last,ss_ (h)	median	23.9	95.4	168	169
	(range)	(4.0–240)	(23.5–239)	(71.5–240)	(72.4–383)
AUC_0-tau,ss_ (h*ng/mL)	mean (STD)	85.1 (84.1)	175 (79.8)	457 (191)	958 (393)
	median	60.1	163	554	1021
	(range)	(5.5–263)	(93.4–354)	(144–655)	(227–1444)
T_1/2,ss_ (h)	mean (STD)	25.7 ^2^ (24.0)	44.1 (24.2)	67.9 ^3^ (20.6)	51.1 (15.9)
	median	14.6 ^2^	40.6	61.2 ^3^	48.6
	(range)	(2.4–64.4)	(8.9–78.5)	(46.6–99.6)	(31.3–83.8)
CL/F (L/h)	mean (STD)	215 (333)	87.4 (32.0)	74.6 (48.0)	74.7 (61.7)
	median	111	81.5	48.0	52.1
	(range)	(25.3–1200)	(37.6–142)	(40.6–185)	(36.8–234)
Vz/F (L)	mean (STD)	2980 (1606)	4755 (2072)	6020 ^3^ (3552)	4855 (2392)
	median	2843	4033	5139	4595
	(range)	(738–5482)	(1825–7872)	(2945–14,135)	(2311–10,634)
C_av,ss _ (ng/mL)	mean (STD)	3.6 (3.5)	7.4 (3.3)	19.3 (8.1)	39.9 (16.6)
	median	2.5	6.9	23.4	42.9
	(range)	(0.2–11.0)	(4.0–14.8)	(6.2–27.9)	(9.3–60.9)
C_trough_ (ng/mL)	mean (STD)	1.40 (1.79)	2.74 (1.61)	7.70 (3.71)	20.5 (12.2)
	median	0.9	2.5	8.8	21.3
	(range)	(0–5.3)	(0.7–5.8)	(2.3–12.3)	(3.4–39.7)
Fluctuation	mean (STD)	6.54 (2.28)	4.66 (1.44)	3.94 (1.41)	3.53 (1.37)
	median	5.6	5.0	3.4	3.3
	(range)	(4.5–11.8)	(2.5–6.8)	(2.2–6.8)	(2.0–6.7)

^1^ *n* = 11; ^2^ *n* = 10; ^3^ *n* = 8; STD, standard deviation; CL/F, apparent clearance; Vz/F, apparent volume of distribution.

**Table 4:** Mean (standard deviation) and median (range) plasma pharmacokinetic parameters for 7-hydroxymitragynine after increasing single (SD) and 15 multiple doses (MD) of mitragynine in dried kratom leaf powder.

		Cohort 1 6.65 mg	Cohort 2 13.3 mg	Cohort 3 26.6 mg	Cohort 4 53.2 mg
Single dose		*n* = 12	*n* = 12	*n* = 12	*n* = 13
C_max_ (ng/mL)	mean (STD)	3.6 (1.9)	6.6 (1.1)	10.8 (3.7)	22.7 (7.7)
	median	3.6	7.0	11.0	21.7
	(range)	(1.5–8.6)	(4.8–7.8)	(4.3–16.0)	(12.5–38.6)
T_max_ (h)	median	1.2	1.5	1.8	1.7
	(range)	(0.8–2.7)	(1.0–2.3)	(0.8–5.0)	(1.0–2.3)
T_last_ (h)	median	5.0	9.0	12.0	23.3
	(range)	(3.5–9.0)	(6.0–12.1)	(0.75–12.0)	(9.0–71.6)
AUC_0-24_ (h*ng/mL)	mean (STD)	9.7 (5.4)	25.8 (6.7)	47.7 (22.4)	125 (69.7)
	median	9.0	25.3	54.3	115
	(range)	(2.9–19.3)	(14.7–37.6)	(0.7–78.8)	(40.2–319)
AUC_0-Tlast_ (h*ng/mL)	mean (STD)	9.7 (5.4)	25.8 (6.7)	47.7 (22.4)	130 (84.2)
	median	9.0	25.3	54.3	115
	(range)	(2.9–19.3)	(14.7–37.6)	(0.7–78.8)	(40.2–380)
AUC_0-inf_ (h*ng/mL)	mean (STD)	11.2 (5.6)	28.8 (7.6)	58.2 ^1^ (20.4)	136 (86.6)
	median	10.5	28.0	64.5 ^1^	123
	(range)	(4.1–21.0)	(16.4–41.8)	(27.6–85.7)	(41.6–391)
AUC_Tlast_/	median	0.85	0.90	0.91	0.96
AUC_0-inf_	(range)	(0.71–0.92)	(0.87–0.92)	(0.81–0.95)	(0.93–0.97)
T_1/2 _ (h)	mean (STD)	1.7 (0.5)	2.9 (0.9)	3.2 ^1^ (0.8)	4.7 (2.7)
	median	1.7	2.7	3.4	4.0
	(range)	(1.0–2.4)	(1.5–4.5)	(1.9–4.5)	(1.7–11.4)
Multiple doses		*n* = 11	*n* = 8	*n* = 9	*n* = 9
C_max,ss _ (ng/mL)	mean (STD)	3.2 (1.5)	5.9 (1.5)	11.3 (3.5)	21.8 (6.1)
	median	3.3	5.6	12.0	20.9
	(range)	(1.4–5.6)	(4.3–8.1)	(6.9–17.4)	(13.3–31.7)
T_max,ss _ (h)	median	1.3	1.7	1.7	2.00
	(range)	(0.8–3.0)	(1.0–4.0)	(1.3–3.0)	(1.3–3.5)
T_last,ss _ (h)	median	5.0	12.0	23.5	48.3
	(range)	(3.5–12.0)	(6.0–23.9)	(9.0–47.6)	(9.0–121)
AUC_0-tau,ss_ (h*ng/mL)	mean (STD)	10.1 (6.8)	28.0 (13.7)	67.8 (31.3)	133 (45.0)
	median	7.2	27.3	67.7	128
	(range)	(3.2–22.9)	(13.5–55.7)	(25.0–119)	(105–161)
T_1/2,ss _ (h)	mean (STD)	2.4 (1.3)	3.8 (2.3)	8.3 (5.0)	24.7 (24.7)
	median	1.9	3.2	9.0	9.1
	(range)	(1.1–5.3)	(1.5–8.4)	(2.1–16.7)	(2.2–71.6)
C_ave,ss _ (ng/mL)	mean (STD)	0.4 (0.3)	1.2 (0.6)	2.9 (1.3)	5.5 (1.9)
	median	0.3	1.1	2.9	5.4
	(range)	(0.1–1.0)	(0.6–2.3)	(1.0–5.0)	(1.6–8.2)
C_trough_ (ng/mL)	mean (STD)	0.0 (0.0)	0.1 (0.3)	0.6 (0.6)	1.7 (1.0)
	median	0	0	0.6	1.6
	(range)	(0–0)	(0–0.7)	(0–1.3)	(0–3.2)
Fluctuation	mean (STD)	8.8 (2.2)	5.6 (2.1)	4.3 (1.8)	4.1 (1.7)
	median	9.8	5.3	3.4	3.7
	(range)	(5.9–11.3)	(2.7–8.9)	(2.7–6.9)	(2.3–8.2)

^1^ *n* = 11; STD, standard deviation.

## Data Availability

The data presented in this study are available upon request from Ramsey Atallah, Della Terra Pharmaceuticals.
